# Transcatheter Closure of Patent Ductus Arteriosus in Adults

**DOI:** 10.1007/s11886-026-02395-z

**Published:** 2026-07-29

**Authors:** Ka-Chun Un, Pramod Sagar, Ronak Sheth, Ah-Young Kim, Anh-Quoc Dao, Mario Carminati

**Affiliations:** 1 Cardiology Division, Department of Medicine, School of Clinical Medicine, Li Ka Shing Faculty of Medicine, The University of Hong Kong, Hong Kong SAR, China; 2 Cardiology Division, Department of Medicine, Queen Mary Hospital, The University of Hong Kong, Hong Kong SAR, China; 3 Department of Pediatric Cardiology, Institute of Cardiovascular Diseases, Madras Medical Mission, Chennai, Tamil Nadu India; 4 Department of Pediatric Cardiology, Fortis Pediatric and Congenital Heart Centre, Mumbai, Maharashtra India; 5 Department of Pediatrics, Division of Pediatric Cardiology, University of California-Davis, 2521 Stockton Boulevard, Sacramento, CA 95817 USA; 6 Cardiovascular Center, University Medical Center, University of Medicine and Pharmacy, Ho Chi Minh City, Vietnam; 7 Pediatric and Adult Congenital Disease Heart Centre, IRCCS Policlinico San Donato, Piazza E. Malan 2, San Donato Milanese, 20097 Italy

**Keywords:** Patent ductus arteriosus, Adult congenital heart disease, Pulmonary hypertension, Transcatheter closure, Multi-centre registry

## Abstract

**Purpose of Review:**

Data on transcatheter closure of patent ductus arteriosus (PDA) have been widely reported in pediatric but not adult patients. This paper serves as an overview of the currently available evidence and supplements it with additional data from our multi-centre registry.

**Recent Findings:**

Transcatheter closure of PDA is safe and effective in adults, with up to 90–100% success rate. Our multi-centre registry shows similar findings with low risk of complications. Yet there are special anatomical, imaging and technical considerations compared to the pediatric population, and the common presence of pulmonary arterial hypertension complicates clinical decision-making.

**Summary:**

Transcatheter closure is less commonly performed in the adult population and is not as straightforward as commonly perceived. Given the paucity of studies reported in the current literature, further large-scale trials are needed to determine the optimal approach and long-term outcomes.

## Introduction

The ductus arteriosus (DA) is a vascular connection between the descending aorta (DAo) and pulmonary artery (PA) that exists in normal fetal circulation. It is important in directing blood away from the lungs to achieve systemic oxygenation before birth. After birth, the lung becomes functional for gas exchange with the drop in pulmonary vascular resistance, and the DA is no longer needed. In normal term infants, the DA closes in > 90% by 48 h [1], while in preterm infants, the immaturity of the DA sometimes leads to delayed closure [2]. In some individuals, the DA remains patent (PDA) even into childhood or adult life.

## Clinical Presentation and Indication for Closure

The clinical presentation of PDA in adults depends largely on the size of the duct. In the case of a large duct, the significant amount of blood from the left-to-right shunting leads to histological changes of the vessel, raised pulmonary vascular resistance and pulmonary arterial hypertension (PAH). In more severe cases, the pulmonary vascular resistance may exceed systemic vascular resistance, leading to reversed shunt and systemic cyanosis, known as Eisenmenger Syndrome [3]. In less severe cases, the increased pulmonary blood flow causes an increase in pulmonary venous return, leading to left atrial and left ventricular (LV) dilatation [4]. When the PDA is small, it can be clinically silent and without any haemodynamic consequence. However, the flow turbulence may predispose patients to infective endocarditis (IE), the incidence of which is 1% in various studies [5]. As a result, PDA in an adult is not considered a benign entity, with a mortality rate of about 1.8% per year [6].

According to the 2020 European Society of Cardiology Guideline, closure of the PDA is suggested when there is evidence of LV volume overload in the absence of pulmonary arterial hypertension; when there is pulmonary arterial hypertension but still a significant left-to-right shunt with Qp: Qs > 1.5 and pulmonary vascular resistance (PVR) 3–5 Woods Units (WU); and for selected cases when PVR is more than 5 WU. The current guideline supports closure of haemodynamically significant PDAs only, although historically, prophylaxis for IE has been considered as an indication for closure as well [7]. Previously, all PDAs with a murmur were advised closure to prevent endarteritis, even small ducts [8].

## Technical Consideration for Closure

### Anatomical Considerations

**Fig. 1:**
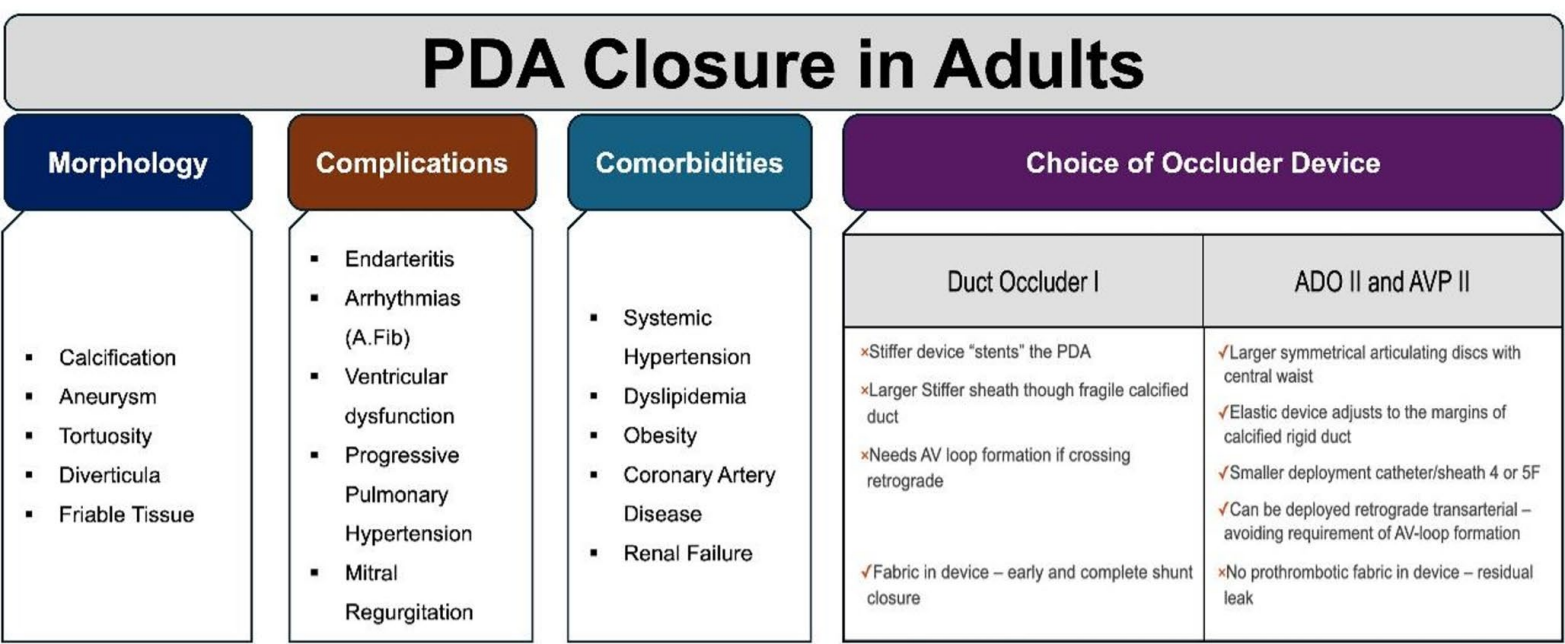
Important considerations in procedural planning for transcatheter closure of Patent Ductus Arteriosus (PDA) in adults – Morphologic factors, Complications, Co-morbidities and Device choice. ADOII – Amplatzer Duct Occluder II, AVPII – Amplatzer Vascular Plug II, AV loop – arteriovenous loop. *Red checkmarks* indicate advantage and *red crosses* indicate disadvantage of using the device

The procedural complication rate in adult PDA closure is reported to be 11% [9, 10]. The degree of calcification and atherosclerotic changes of the PDA as well as vascular walls increases the incidence of procedural complications (Fig. 1). Surgical closure of such PDA is associated with serious complications, and cardiopulmonary bypass is often needed.

Calcification in the wall of the PDA may result in difficulty crossing the duct antegrade from the pulmonary end, as well as challenging negotiation of the device and delivery sheath across the ductus. Careful planning and procedural modification may be needed according to the individual anatomy [9]. Significant aortic calcification may result in distal cholesterol embolization during manipulation and lead to renal or limb ischemia .

### Imaging Considerations

Transthoracic echocardiography remains the primary imaging modality for the evaluation of PDA in adults despite the usual challenges of suboptimal adult echocardiographic windows and poor visualization of calcified structures due to artifact. Echocardiography provides crucial information to quantify the shunt, assess haemodynamics and associated abnormalities, as well as assess PDA morphology and sizing. Cardiac Magnetic Resonance Imaging could assess left ventricular volumes, shunt fractions and associated lesions. CT aortography overcomes the limitations of echocardiography in patients with suboptimal sonographic windows, helps characterize the morphology of the ductus, defines the size and angiographic classification of PDA morphologic subtype, studies calcification of the ductal tissue or the surrounding aortic wall, and excludes other causes of continuous murmurs (including coronary fistulae and aortopulmonary collaterals) [11, 12]. Intravascular ultrasound has also been employed for sizing and procedural guidance [13].

### Transcatheter Closure Steps

#### Sizing

Accurate sizing of the PDA is the most crucial aspect of adult PDA transcatheter closure for prevention of complications like residual shunt or device embolization. In adults, the larger vessel sizes with higher blood flow and contrast streaming effects in the aorta lead to poor opacification and suboptimal delineation of vascular structures by conventional angiography; thus, accurate sizing of PDA in adults may be difficult with conventional techniques.

Balloon sizing of the PDA [14] can be used when angiographic measurements are difficult. After forming an arteriovenous circuit across the PDA, a very compliant balloon catheter (e.g. Amplatzer sizing balloon, usually 18–24 mm, Abbott Structural Heart, USA) is parked through the venous access in the descending aorta. It is then inflated with diluted contrast, up to about two-thirds of maximum volume with pressure usually < 1 atm. This inflated balloon is then gradually pulled back from the DAo across the ductus into the PA while performing a cine-acquisition in the most non-foreshortened view. The compliant balloon approximates the ductus wall as it passes across it, thus giving a good delineation of ductal morphology as well as an accurate estimate of the size, which correlates well with CT-derived measurements [15].

Angiographic sizing of the DA by conventional and CT angiography also does not consider the natural elasticity of ductal tissue and tends to underestimate the distended ductal diameter. Therefore, systematic oversizing is often required to avoid residual shunt and device embolization [16]. The ductus is also notorious for having spasm during catheterization, which may lead to systematic undersizing by angiography alone [17, 18]. As a result, the balloon pull-through technique provides “dynamic sizing” that accounts for ductal tissue elasticity and thus increases procedural success and safety [16].

### Device Choices

#### Amplatzer Duct Occluders for Adult PDA closure

The greatest experience for closure of adult PDA is with the use of Amplatzer Ductal Occluders (Abbott Structural Heart, USA), which are suitable for a wide variety of morphologies and have excellent procedural safety and long-term results [12, 19].

#### Specific Duct Occluders / Vascular Plugs

Occlutech Duct Occluders (Occlutech, Switzerland) have potential design advantages, including a larger width at the PA end, a longer length for better stability, and a prominent screw attachment at the PA end facilitating easy snaring if retrieval is necessary. Some specific anatomies may require a relatively longer duct occluder device for successful closure [9, 20, 21]. The Occlutech Occluder and Cera Duct Occluders (Lifetech Scientific, China) are available in larger sizes up to 26/24 mm and are useful for larger ducts. The Amplatzer Ductal Occluder II (ADO II) device is useful for ducts up to size < 5.5 mm. Amplatzer Vascular Plugs (AVP) II and IV are useful for long tubular ducts, especially small ducts that cannot be crossed antegrade, as these can be delivered retrograde from the arterial end [12]. These are the devices of choice in older populations with deformed, fragile and heavily calcified ducts, which are often challenging via the antegrade transvenous approach [10] (Fig. 1).

### Atrial Septal Occluders for PDA closure

**Fig. 2:**
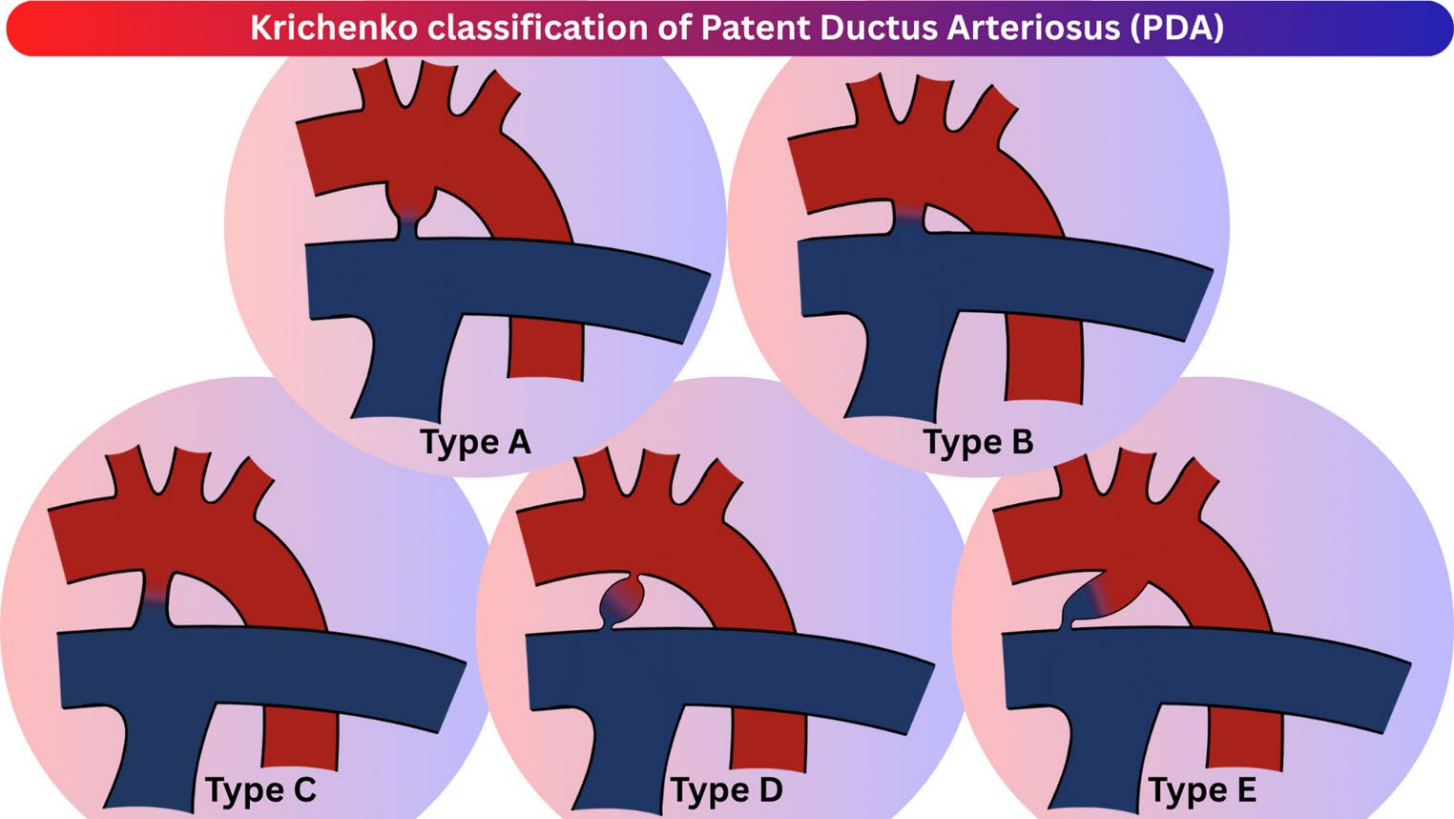
Kirchenko classification of PDA morphologies. Type A: Conical shape with prominent aortic ampulla and constriction at pulmonary artery end, Type B: Short and wide (window type), Type C: Long tubular ductus without constriction, Type D: With multiple constriction (complex type), Type E: Elongated ductus with a constriction remote from the anterior edge of the trachea

Garcia-Montes et al. [22] described one of the largest series of older patients with large hypertensive PDA closed using Amplatzer Septal Occluders (ASO, Abbott Structural Heart, USA). In these patients with large hypertensive ducts, there is a significant risk of inadvertent undersizing and device embolization (35%). Using the ASO device, a mild to moderate residual shunt was present in more than 80% of patients. However, most of them had a gradual reduction in residual shunt and good reduction of PA pressures on follow-up [12, 22]. Atrial septal occluders in general are suitable for wide and short PDA [10] (Fig. 2).

### Other Occlusion Devices

The Muscular Ventricular Septal Defect (VSD) Occluder (MVSDO, Abbott Structural Heart, USA) is the device of choice for large ducts with significant pulmonary hypertension [10, 12, 16, 23]. MVSDO, ASO and Gianturco-Grifka Vascular Occlusion devices are more suitable in specific scenarios with large ducts when conventional duct occluders are not suitable [24].

**Fig. 3:**
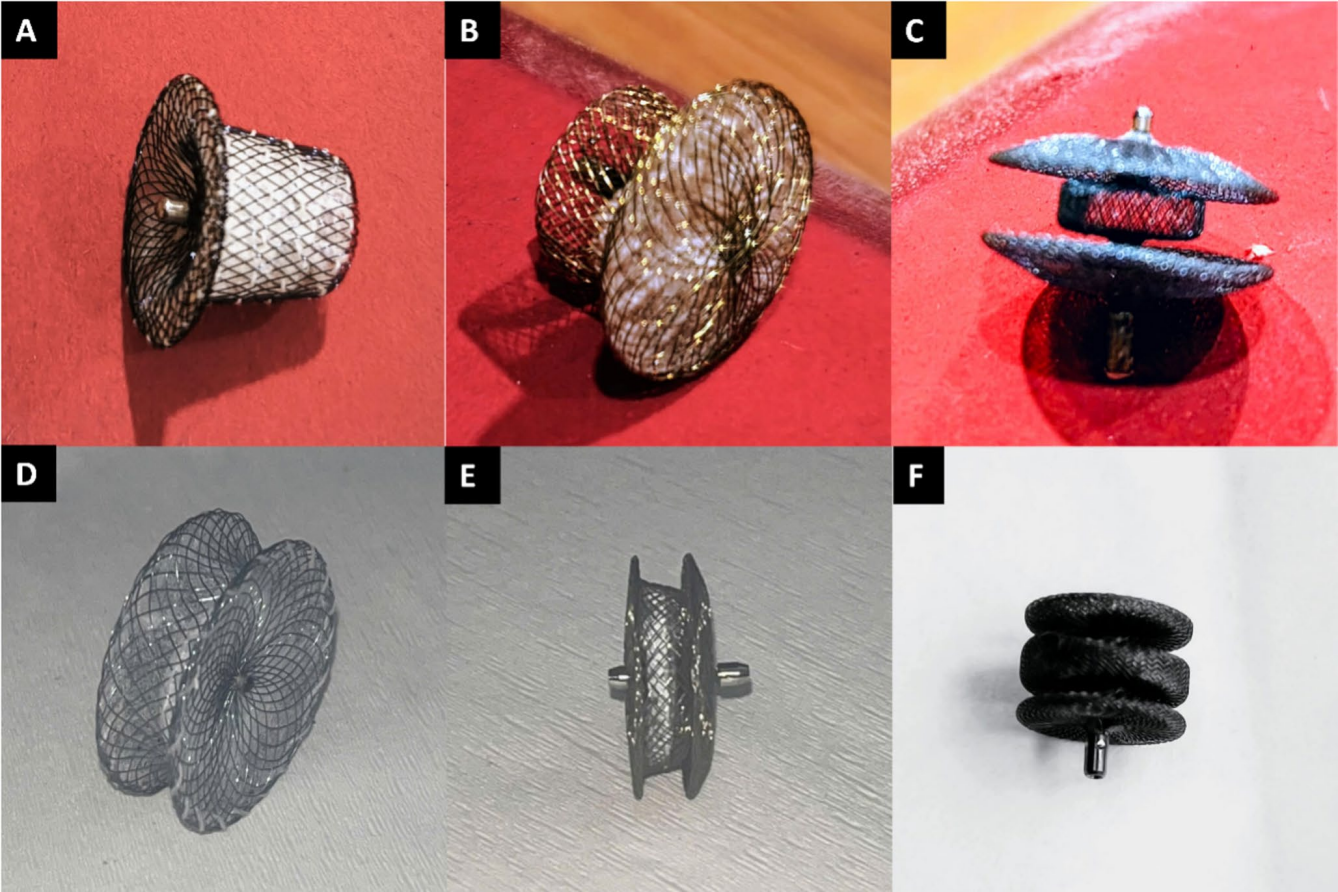
Examples of commonly used occluders for PDA closure. **A**) Amplatzer Duct Occluder; **B**) Occlutech PDA Occluder; **C**) Amplatzer Duct Occluder II; **D**) Amplatzer Muscular VSD Occluder; **E**) KONAR-MF VSD Occluder; **F**) Amplatzer Vascular Plug II

NitOcclud Coils (pfm medical, Germany) are safe and effective for PDA closure in various ductal sizes and morphologies, especially in small and moderate-sized ducts. Even in larger ducts, procedural success is high, albeit with a higher incidence of residual shunt at one-year follow-up; however, this seems to be easily amenable to additional coils or secondary devices [25]. Figure 3 shows the common occluders used for PDA closure.

### Thoracic Endovascular Aortic Repair (TEVAR) for PDA closure

TEVAR, which has traditionally been used in thoracic aortic pathologies such as dissection or aneurysm, has also been used for older patients with calcified ductus [26–28]. By direct exclusion of the ductus, TEVAR can be an alternative transcatheter technique for complicated PDA in the elderly population as a safe option when other devices are not suitable.

## Closure in Special Patient Groups

### Special Considerations in Older Population with PDA (> 55 years)

The incidence of pre-existing comorbidities is highest among the older population. A large series of PDA closure in patients > 55 years of age had nearly a 50% incidence of pronounced calcification, with 70% having comorbid hypertension, 66% pulmonary hypertension, 50% atrial fibrillation, 20% coronary artery disease, and 6% renal failure [10].

As a result, the incidence of procedural complications is significant in this subgroup, including groin hematomas, hypertensive urgency requiring afterload reduction, inadvertent undersizing causing device embolization, residual shunt, hemolysis, and arrhythmias (especially new-onset atrial fibrillation).

### PDA with Pulmonary Hypertension in Adults

The exact prevalence of PAH in adults with PDA is not known due to the lack of adult congenital heart disease (ACHD) registries worldwide [29]. A PDA that is non-restrictive and large enough to cause significant pulmonary hypertension is likely to be detected in infancy or early childhood; if undiagnosed, it is likely to develop significant pulmonary vascular disease resulting in right-to-left shunt with hypoxia in the lower half of the body. However, in some cases large PDAs can still have pertinent left-to-right shunts. Moderate-sized PDAs are likely to have persistent left-to-right shunts even in adult life and can cause mild to moderate pulmonary hypertension, while small restrictive PDAs can remain asymptomatic in adult life without accompanying pulmonary hypertension. Small PDAs can be just coincidental in some cases of primary pulmonary hypertension and can decompress the right ventricle. The proportion of different types of PAH in the operable range and Eisenmenger syndrome is unknown. In the Japanese adult congenital heart disease registry, the prevalence of PAH in PDA was 4.3%, and 2.7% had Eisenmenger syndrome [30].

Determination of the severity of pulmonary hypertension and deciding on operative feasibility is essential in moderate to large PDAs, particularly in adults. Although measurements of the shunt ratio between pulmonary and systemic circulation (Qp: Qs), indexed pulmonary vascular resistance (PVRi), and the ratio of pulmonary to systemic vascular resistance (PVR: SVR) are commonly used in atrial and ventricular septal defects, they are not uniformly collected for PDA due to challenges accounting for differential shunting to the right and left pulmonary arteries, leading to variable saturation in the branch pulmonary arteries [30]. The 2018 American Heart Association/American College of Cardiology ACHD guidelines suggest a Class I recommendation for closure in PDA with net left-to-right shunt and PA systolic pressure less than 50% of systemic pressure and PVR less than one-third of SVR, and a Class IIb recommendation for net left-to-right shunt despite PA systolic pressure (PASP) more than 50% of systemic and PVR more than one-third of SVR. Net right-to-left shunt with PASP more than two-thirds of systemic and PVR more than two-thirds of SVR is a contraindication [31]. The European Society of Cardiology ACHD guidelines suggest a Class I recommendation for closure only when Qp: Qs is > 1.5 and PVR is < 3 WU, Class IIa for Qp: Qs > 1.5 and PVR of 3–5 WU, and Class IIb for Qp: Qs > 1.5 with PVR > 5 WU. Eisenmenger syndrome with lower limb oxygen saturation < 90% is considered a contraindication [32]. Both guidelines do not provide recommendations for borderline cases. Furthermore, the evidence for the recommendations is poor in both guidelines (Level of evidence C).

PDA occlusion testing is often used in adults with PDA and PAH to determine operability. Initially, balloon occlusion testing was attempted with simultaneous PA and aortic pressure measurement for assessment of operability in the surgical era and was found to be useful [33–35]. However, recent studies often use device occlusion for a few minutes, followed by a decision to release or retrieve the device depending on the reduction in PA pressure [18, 36, 37]. The cutoff value used for operability in terms of reduction in PA pressure is not uniform. In a study by Zhang et al. [36] evaluating predictors of freedom from post-procedural PAH, a 50% reduction in systolic PA pressure was found to be the sole determinant on multivariate analysis, although various parameters including higher LV dimensions, lower PA pressures at baseline, PVR, and PVR: SVR ratio were significant on univariate analysis.

However, persistent PAH is a major concern after closure of PDA, ranging from 13% to 31%, and decisions on complete closure should be made cautiously with meticulous follow-up [36–38]. Although partial closure with fenestration is recommended for shunt lesions with borderline operability by the 2019 European Pediatric Pulmonary Vascular Disease Network (EPPVDN) guidelines and is a safe option, partial closure of PDA is difficult and not routinely practiced either surgically or by transcatheter means [39]. Only three cases of partial device closure of PDA have been reported in children and adolescents since the first report by Singhi et al. in 2016 using a coronary stent within a muscular VSD occluder [40–42]. Although there are no reports of the same in adults, one case in our multi-centre study had partial closure using a stent in a muscular VSD occluder. However, long-term patency data for these fenestrations are not available, though this can be considered an option in selected cases with close follow-up. The “treat to close” strategy, widely evaluated in adults with atrial septal defect, has not been evaluated in adults with PDA and PAH.

### PDA and Pregnancy

PDA in women of reproductive age poses significant challenges during pregnancy due to the physiological cardiovascular adaptations that occur. These include increases in blood volume, heart rate, and cardiac output, which can exacerbate left-to-right shunting through the ductus, potentially resulting in volume overload, atrial arrhythmias, and PAH [43, 44].

In cases of small, haemodynamically insignificant PDA, pregnancy is typically well tolerated, and spontaneous vaginal delivery can be pursued. However, moderate to large PDAs, especially those associated with left heart chamber enlargement or elevated pulmonary artery pressures, are associated with increased maternal and fetal risk. Importantly, severe PAH is considered a contraindication to pregnancy due to a maternal mortality rate that may exceed 30–50% [45].

Current guidelines recommend that women with significant PDA undergo comprehensive preconception evaluation, including echocardiographic assessment of shunt severity, ventricular function, and pulmonary pressures. Closure of the PDA before conception is advised when there is evidence of left heart volume overload or borderline pulmonary pressures [10].

A multidisciplinary approach involving cardiologists, maternal-fetal medicine specialists, and anaesthesiologists is essential during both gestation and delivery planning [46]. Vaginal delivery is preferred in clinically stable patients, while caesarean section may be necessary for obstetric or severe cardiac indications. Table 1 summarizes the currently available evidence on PDA closure in adults [9, 10, 12, 47–51].

**Table 1:** Summary of Studies in Transcatheter Closure of PDA in Adults

Study/ Study period	Inclusion Criteria	Total Cases/Age	Narrowest PDA Diameter	Calcification/ Residual PDA	Symptomatic/Prior endocarditis	PAH/mPAP (mmHg)	Transvenous crossing/ Sizing	Device Used	Procedural success/Major complication	Residual on >3 months/ Persistent PAH on follow-up	Comments
Wilson et al. [12] 2001-2017	Age>18 yrs	141/41 (18-82)	4 (1-14)	- / 2.1% (post device)	45%/1%	mPAP >35: 6.4% /23 (13-68)	85%/Balloon size-1.4%	ADO-97%, MVSDO-2.1%, ASO-0.7%, AVP 4-0.7%	99%	1.40%/ RVSP>50 mmHg: 1.4%	Double disc device for PAH
Alkashkari et al. [9] 2009-2018	Adults ≥18 yrs	27/24 (18-57)	4.1±2.1	0/0	96%/3.70%	-/22 (16-36)	92.50%/Angiography	ADO-92.6%/ NitOcclud Coils -7.4%	100%/ Cholesterol embolization, AKI in 1	0/0	0/0
P Sudhakar et al. [47] 2011-2017	Age ≥10 yrs, stratified into <18 and >18 yrs	37 (>18Yr)/ 31±10	4.9 (4-8.2)	0/0	91.90%/0	-/26 (22-33)	91.90%/Angiography	ADO-97.3%, Coil-2.7%	100%	0/0	-
Gamboa et al. [48] 1992-2008	Adults ≥16 yrs	23/24 (16–75)	3.5 (1.8–5.8)	8.70%/0	-	-/15 (9–72)	-	Rashkind-30.4%, ADO-52.1%, NitOcclud Coils-8.7%	95.60%	4.30%/0	Rashkind (*n*=7), ADO (*n*=10), Nit-Occlud (*n*=6)
Hong et al. [49] 2002-2002	Adults ≥18 yrs	41/ 35.6 (18–70.7)	-	-	-	-	-	ADO-100%	97.60%	2.5	-
Galeczka et al. [10] 1993-2018	Adults >55yrs	33/63 (56-85)	4.2 (1.5-7)	51.50%	90.90%/0	mPAP >35 mm Hg: 18.1% /30.5 (12-55)	-/-Balloon sizing-21%, CT-12.1%	DO-51.5%, ADO 2-21.2%, ADO2 AS- 6.1%, MVSDO- 9.1%, AVP2-6.1%, ASD devices-9.1%	100%/ Embolization in 1	3%/0	MVSDO for PAH, MR and NYHA class improved on follow up
Bentham et al. [50] 2000-2012	Age>16 yrs	31/ 36.8 (16-80)	3 (2-6)	-	-	-	67.70%/-	ADO-80.6%, Coil-19.4%	100%	3.2	-
Bonhoeffer et al. [51] 1990-1992	Adults with PDA	21/ 40 (19–62)	4.3 (3-9)	33.30%/14.3% (post-surgery)	28.60%/0	mPAP>35 mm Hg: 19.1%	-	Rashkind-100%	90.5%, surgery for residual and hemolysis in 2	-	Established safety of transfemoral approach Higher residual in calcified lesion

*PDA* patent ductus arteriosus, *PAH* pulmonary arterial hypertension, *mPAP* mean pulmonary artery pressure, *PA* pulmonary artery, *ADO* Amplatzer Duct Occluder, *MVSDO* Muscular Ventricular Septal Occluder, *ASO*: Amplatzer Septal Occluder, *AVP* Amplatzer Vascular Plug, *RVSP* right ventricular systolic pressure, *MR* mitral regurgitation, *NYHA* New York Heart Association

## Long-term Data after Closure

### Remodelling Following PDA Closure

Most patients who have immediate post-procedural residual shunts show complete abolition of the residual shunt by 6 months of follow-up. Most of the left ventricular (LV) remodeling with reduction in LV size occurs in the first 6 months after the procedure, and there are often co-existing cardiovascular comorbidities in those who have incomplete improvement in LV dilatation (e.g. coexisting hypertensive or ischemic cardiomyopathy) [9].

### Left Ventricular Dysfunction after Patent Ductus Arteriosus Closure in Adults

Long-standing left-to-right shunting leads to chronic LV volume overload. This causes adaptive LV dilation and remodeling, which can mask subclinical myocardial dysfunction. After PDA closure, particularly in older adults, the sudden haemodynamic changes may precipitate or unmask both systolic and diastolic LV dysfunction [52–54].

Systolic dysfunction may occur post-closure due to the abrupt reduction in preload and increased afterload. This shift can reveal impaired contractility that was previously compensated by volume loading. The reported incidence ranges from 10% to 25%, with most cases being transient. Risk factors include pre-closure LV ejection fraction (LVEF) < 60%, large PDA size (> 6 mm), LV end-diastolic diameter > 56–60 mm, and elevated pulmonary artery pressure. Some patients recover LV systolic function within months, but persistent dysfunction may occur in those with advanced myocardial remodeling [53, 55].

Diastolic dysfunction, while less frequently studied, is also clinically relevant. Chronic volume overload can impair LV compliance and relaxation. After PDA closure, elevated LV filling pressures may persist or worsen in susceptible individuals, particularly older adults or those with existing hypertrophy or fibrosis. Diastolic dysfunction may contribute to persistent symptoms such as dyspnea, even when systolic function appears normal [55, 56]. Despite its significance, diastolic dysfunction is often under-assessed in adult PDA studies. Comprehensive echocardiographic evaluation, including tissue Doppler and strain imaging, should be considered before and after closure [56–58]. Further prospective studies are needed to clarify the long-term prevalence, mechanisms, and management strategies for LV dysfunction following PDA closure in adults.

## Multi-centre Registry on Adult PDA Device Closure

A retrospective multi-centre study was conducted in patients older than 18 years who underwent PDA device closure at four centres in three countries (Queen Mary Hospital, Hong Kong, China; Madras Medical Mission, India; Fortis Pediatric and Congenital Heart Centre, Mumbai, India; and University of Medicine and Pharmacy at Ho Chi Minh City, Vietnam). A total of 66 cases from 2011 to 2024 were included, with six patients excluded because of limited data.

The median age of patients was 38 years, with a predominance of female patients. Symptomatic presentation with effort intolerance or dyspnea was reported in 42.4%, with the majority in NYHA Class II. Comorbidities included systemic hypertension (16.7%), diabetes (7.6%), dyslipidemia (4.5%), and chronic kidney disease (1.5%). A continuous murmur was noted in 71.2% of cases. Pre-procedural imaging predominantly relied on transthoracic echocardiography (87.9%), with limited use of cardiac magnetic resonance imaging (CMR) and CT.

Krichenko type A (89.1%) was the most common type, with a median PDA diameter at the pulmonary artery end of 5 mm. Calcification was identified in 10.6% of patients, most of whom were older (median age 63 years); aneurysmal changes were not seen. Pulmonary hypertension was noted in 53.8%, with moderate to severe PAH in 12 cases (Table 2).

**Table 2:** Summary of Multi-centre Registry of Transcatheter Closure of PDA in Adults

Multi-Centre Registry	
Parameter	N-66 (%)
Age (yr)*	38 (18-76)
Female gender^†^	48 (72.7)
Weight (Kg)*	52.5 (32-92)
Height (cm)*	155 (140-180)
Symptomatic^†^ Effort intolerance/Dyspnea	31 (46.9)
NYHA class^†^	
II	28 (42.4)
III	2 (3.0)
IV	1 (1.5)
Past Infective Endocarditis^†^	1 (1.5)
Systemic Hypertension^†^	11 (16.7)
Diabetes Mellitus^†^	5 (7.6)
Dyslipidaemia^†^	3 (4.5)
Chronic kidney disease^†^	1 (1.5)
Situs inversus^†^	1 (1.5)
Interrupted inferior vena cava^†^	2 (3.0)
Continuous murmur^†^	47 (71.2)
Cardio-thoracic Ratio (%)*	52.5 (42-75)
Pre procedural imaging^†^	
Only TTE	58 (87.9)
CMR	5 (7.6)
CTA	3 (4.5)
PDA type Krichenko (n-55)^†^	
Type A	49 (89.1)
Type B	2 (3.6)
Type C	1 (1.8)
Type D	1 (1.8)
Tortuous	1 (1.8)
Post surgical residual PDA	1 (1.8)
PDA diameter at PA end (mm)*	5 (1-24)
Calcification in PDA^†^	7 (10.6)
Age in those with calcification*	63 (50-73)
Aneurysm^†^	0
Pulmonary hypertension (n-52)^†^	
No	24 (46.2)
Mild	6 (11.5)
Moderate	10 (19.2)
Severe	12 (23.1)
Left Ventricular End-diastolic Diameter (mm)*	54 (37-78)
Left Ventricular End-systolic Diameter (mm)*	34 (26-57)
Left Ventricular Ejection Fraction (%)*	60 (26-75)
Severe LV dysfunction (Associated DCM) ^†^	2 (3.0)
Bicuspid aortic valve^†^	3 (4.5)
Severe aortic stenosis	1 (1.5)
Moderate aortic regurgitation	2 (3.0)
Partial anomalous pulmonary venous connection to right atrium^†^	1 (1.5)
Mitral regurgitation^†^	
Mild	18 (27.3)
Moderate	1 (1.5)
Severe	1 (1.5)
Procedural data	N-66 (%)
Anaesthesia^†^	
Local	61 (92.4)
Sedation	2 (3.0)
General anaesthesia	3 (4.5)
Access^†^	
Both artery and vein	60 (90.9)
Only artery	1 (1.5)
Only vein	5 (7.6)
Right internal jugular vein access^†^	1 (1.5)
Oximetry analysis^†^	14 (21.2)
Balloon occlusion for operability^†^	4 (6.1)
Vasoreactivity testing^†^	2 (3.0)
Attempted crossing (n-61)^†^	
Transvenous	38 (62.3)
Transarterial	23 (37.7)
Failed transvenous crossing (n-38)^†^	6 (15.8)
Arterio-venous loop formation (n-61)^†^	26 (42.6)
Coil closure^†^	5 (7.6)
Device closure^†^	61 (92.4)
Type of closure device (n-61) ^†^	
ADO	54 (88.5)
ADO II	1 (1.6)
Muscular VSD Occluder	4 (6.6)
AVP I	1 (1.6)
AVP II	1 (1.6)
Oversizing (%)*	50 (13-300)
Oversizing (mm)*	3 (1-10)
Need of change of device^†^	3 (4.5)
Residual leak^†^	4 (6.1)
Procedural success^†^	66 (100)
Complications^†^	
Femoral artery pseudoaneurysm	1 (1.5)
Hemolysis (self-limiting)	1 (1.5)
Transient LV dysfunction^†^	6 (9.1)

*-Data expressed as median; †-Data expressed as frequency and percentage. NYHA: New York Heart Association; TTE: transthoracic echocardiography; CMR: cardiac magnetic resonance imaging; CTA: computed tomography angiography; PDA: patent ductus arteriosus; PA: pulmonary artery; LV: left ventricular; DCM: dilated cardiomyopathy; ADO: Amplatzer Duct Occluder; ADO II: Amplatzer Duct Occluder II; VSD: ventricular septal defect; AVP: Amplatzer Vascular Plug

**Fig. 4:**
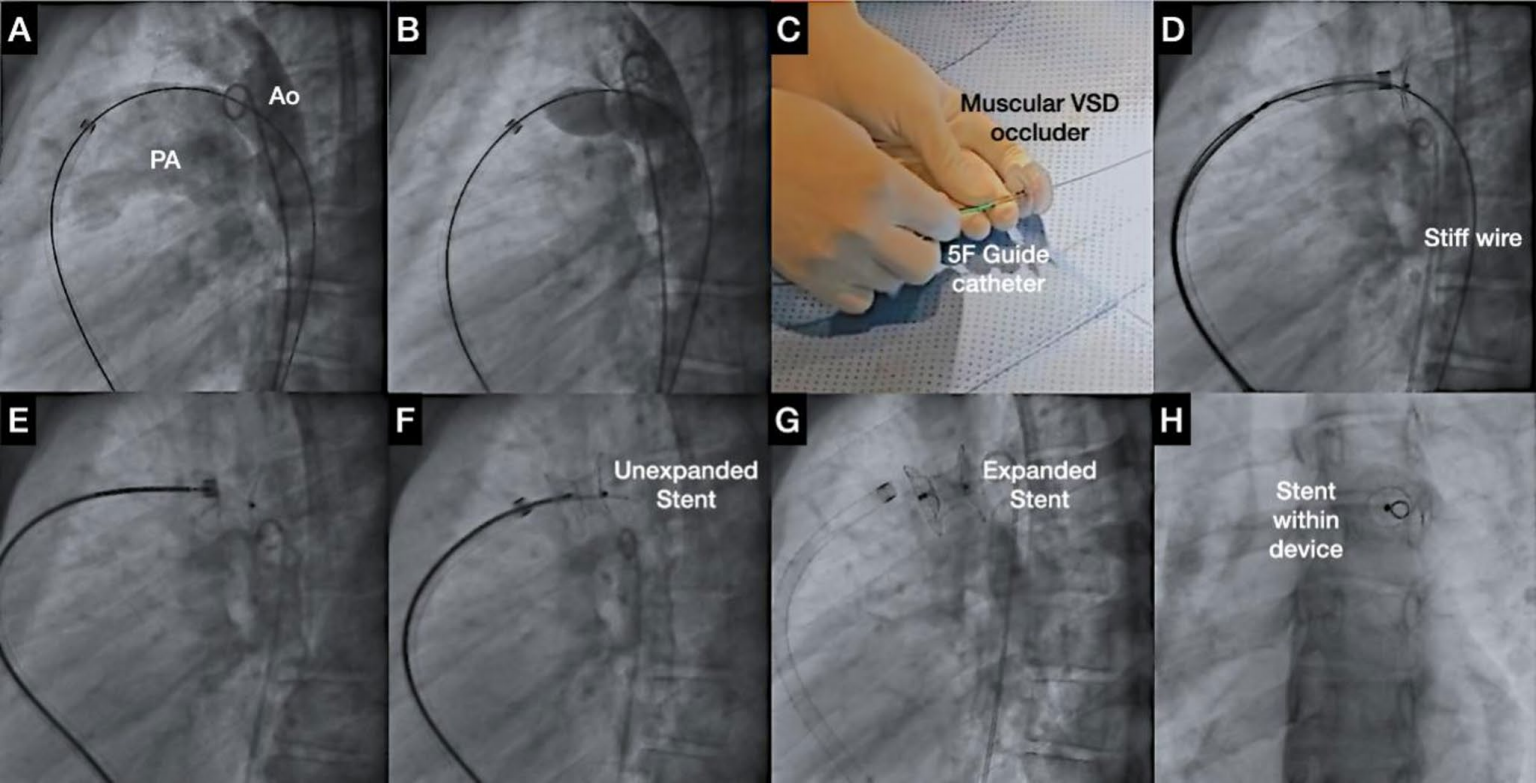
An illustrative case of partial closure of PDA in the registry. **A**) Aortic (Ao) angiogram demonstrating large PDA filling the pulmonary artery (PA); **B**) Complete occlusion of the PDA using 18 × 30 mm Balloon; **C**) Puncturing the 18 mm post infarct muscular VSD occluder and advancing a Judkins Right guide catheter through it; **D**) Advancing the device guide wire assembly through a 14 F sheath and deployment of disc in aorta; **E**) Deployment of device; **F**) 4.5 × 22 mm coronary stent through the guide catheter; **G**) Complete stent deployment; **H**) En-face view of the device with a stent acting as fenestration

Procedures were mostly performed under local anaesthesia (92.4%). Among patients with moderate to severe PAH (*n* = 12), balloon occlusion or vasoreactivity testing was performed in 50% of cases. Transvenous access was the first choice for initial ductal crossing (62.3%) and succeeded in most cases (84.2%). Arteriovenous loop formation was employed in more than 40% of cases. Device-based closure was the dominant strategy (92.4%), with only 5 cases managed with coils. The Amplatzer Ductal Occluder was the most frequently used device (88.5%), followed by other Amplatzer devices and vascular plugs. The overall procedural success rate was 100%. Partial closure with a coronary stent to create a fenestration in a Muscular VSD Occluder was performed in one case with borderline hemodynamics (Fig. 4). Complications were minimal and included one femoral artery pseudoaneurysm and one patient with self-limiting hemolysis (each 1.5%). Transient left ventricular dysfunction occurred in 9.1% of patients.

## Conclusion

Although less commonly performed in the adult population, transcatheter closure of PDA in the adult is not as straightforward as commonly perceived. There are special anatomical, imaging and technical considerations for closure compared to the pediatric population, and the presence of pulmonary arterial hypertension often complicates clinical decision-making. Various types of occluders with differening designs have been well shown to be effective in the pediatric population, but not specifically reported in the adult population. Given the paucity of studies reported in the current literature, pooling multi-centre data with long-term follow-up is required to inform clinicians about the optimal approach and the long-term outcomes of transcatheter closure of PDAs in adults.

## Key References

- Wilson, W.M., et al., Clinical outcomes after percutaneous patent ductus arteriosus closure in adults. Canadian Journal of Cardiology, 2020. 36(6): p. 837-843.
  - ○ Findings from this study represent the largest registry of adults with PDA and demonstrate the safety of device closure.
- Zhang, D.-z., et al., Trial occlusion to assess the risk of persistent pulmonary arterial hypertension after closure of a large patent ductus arteriosus in adolescents and adults with elevated pulmonary artery pressure. Circulation: Cardiovascular Interventions, 2014. 7(4): p. 473-481.
  - ○ Findings from this study illustrate the approach to adult patients with large patent ductus arteriosus and pulmonary arterial hypertension, the role of trial balloon occlusion, and the predictors of adverse outcome.

## Data Availability

Raw data were generated at Queen Mary Hospital, Hong Kong, China; Madras Medical Mission, India; Fortis Pediatric and Congenital Heart Centre, Mumbai, India; and University of Medicine and Pharmacy at Ho Chi Minh City, Vietnam. Derived data supporting the findings of this study are available from the corresponding author upon request.

## Abbreviations

PDA: Patent Ductus Arteriosus
PAH: Pulmonary Arterial Hypertension
CT: Computerized Tomography
ES: Eisenmenger Syndrome
PVR: Pulmonary Vascular Resistance
SVR: Systemic Vascular Resistance
